# Ethanolamine-phosphate phospho-lyase (ETNPPL) contributes to the diagnosis, prognosis, and therapy of hepatocellular carcinoma

**DOI:** 10.7717/peerj.15834

**Published:** 2023-08-21

**Authors:** Yun Zhang, Li Shen, Bojun Wang, Xiaohong Wu

**Affiliations:** 1 Department of General Surgery, The Affiliated Yixing Hospital of Jiangsu University, Yixing, Jiangsu, China; 2 Disinfection Supply Center, The Affiliated Yixing Hospital of Jiangsu University, Yixing, Jiangsu, China; 3 Department of General Surgery, Yixing Fourth People’s Hospital, Yixing, Jiangsu, China

**Keywords:** Hepatocellular carcinoma, ETNPPL, Diagnosis, Prognosis, Migration, Invasion

## Abstract

**Background:**

Hepatocellular carcinoma (HCC) is characterized by high mortality, difficulty in early screening, relapse, and poor prognosis. This study aimed to explore the expression of ethanolamine-phosphate phospho-lyase (ETNPPL) and its clinical significance in HCC.

**Methods:**

Differentially expressed mRNAs were screened using microarray analysis. Functional enrichment was performed using GO (Gene Ontology) and KEGG (Kyoto Encyclopedia of Genes and Genomes) analysis. We used qRT-PCR to measure the expression of ETNPPL in HCC tissues and paired paracarcinoma tissues. A receiver operating characteristic (ROC) curve and Kaplan-Meier curve were conducted to assess the diagnostic and prognostic values. Cell behaviors were evaluated using a scratch test and transwell assay.

**Results:**

The results showed that numerous mRNAs are abnormally expressed in HCC. ETNPPL was decreased in HCC tissues and cells. The area under curve (AUC) of ETNPPL was 0.9089, demonstrating that ETNPPL had diagnostic value. Low expression of ETNPPL was related to poor prognosis for patients with HCC. Moreover, the over-expression of ETNPPL inhibited HCC cell migration and invasion.

**Conclusions:**

In conclusion, downregulated ETNPPL was found in HCC and is related to poor patient prognosis and the promotion of cell metastasis. This suggests that ETNPPL serves both as a promising diagnosis and prognosis biomarker, and a therapy target of HCC.

## Introduction

Hepatocellular carcinoma (HCC) is the most common type of primary liver cancer that occurs in chronic liver disease (Wallace et al., 2015). In the 2020 global cancer incidence and mortality data published by the International Agency for Research on Cancer (IARC), out of 36 cancer types in 185 countries, there were about 900,000 new cases of HCC, accounting for 4.7% of new cancer cases (Sung et al., 2021). The number of new deaths from HCC was 830,000, accounting for 8.3% of total deaths, making it the second most common cause of death. It is expected that the incidence of HCC will exceed 1 million cases by 2025. Therefore, HCC remains a global challenge to human health (Sung et al., 2021). Hepatitis C or B, liver cirrhosis, fatty liver disease, and excessive drinking are risk factors for HCC (Yang et al., 2019). Despite the development of therapeutic strategies for HCC such as surgery or embolic and drug therapies, HCC is still a major cause of cancer death (Hartke, Johnson & Ghabril, 2017). At present, the pathogenesis of HCC is still not fully clarified, and the treatment is traditional, meaning the disease is prone to recurrence and poor prognosis. Moreover, early screening of HCC is hampered by the lack of obvious symptoms and physical changes  (Ren et al., 2020; Ogunwobi et al., 2019). Therefore, it is urgent to explore effective diagnostic and prognostic biomarkers. Distant metastasis of tumor cells is also a main cause of cancer death and reoccurrence (Summers, McDonald & Croucher, 2020), meaning it is of great clinical significance to inhibit tumor cell invasion and migration.

Ethanolamine-phosphate phospho-lyase (ETNPPL), also known as AGXT2L1, is located at 4q25 on the human chromosome and shares 36% sequence identity with ALan-glyoxylate transaminase 2. ETNPPL is frequently expressed in the liver, brain, salivary glands, kidney, and stomach tissues. ETNPPL is a lipid metabolizing enzyme involved in the metabolism of phosphoethanolamine in the cell membrane (Leventoux et al., 2020; Schiroli et al., 2013). Primarily found in brain tissues, change in ETNPPL expression is closely related to human emotions and lipid homeostasis in the brain (White, Ellis & Wolfgang, 2021). Presently, there are few studies on ETNPPL. The existing relevant studies show that the ETNPPL gene is abnormally expressed in multiple types of mental disorders. The study by  McQuillin, Rizig & Gurling (2007) showed that the expression of ETNPPL in mouse brain tissue significantly increased after administration of the mood stabilizer lithium carbonate in a manic mouse model.

Shao & Vawter (2008) found that the expression of ETNPPL significantly increased in the brain tissues of patients with bipolar disorder and schizophrenia compared with normal brain tissues. Accumulating evidence has demonstrated that the expression of ETNPPL occurs in numerous malignancies, such as primary glioblastoma, colorectal cancer, gastric cancer, pancreatic cancer, and HCC (González-García et al., 2020; Deng et al., 2020; Ding et al., 2016). Deng et al. (2020) found that ETNPPL can regulate phosphatidylinositol and phosphatidylserine metabolism in cancer tissues, and knockdown of ETNPPL expression can induce autophagy in colorectal cancer cells. Ding et al. (2016) first found that ETNPPL expression is decreased in HCC and its low expression suggests poor prognosis. Moreover, ETNPPL plays a crucial role in aberrant adipogenesis of HCC tissues. However, the more detailed function of AGXT2L1 remains to be elucidated, especially in the field of HCC.

In this study, we predicted the dysregulation of mRNAs in HCC. ETNPPL is a downregulated mRNA in HCC, and is identified as a diagnosis and prognosis biomarker. Moreover, an absence of ETNPPL promoted HCC cell migration and invasion. The findings suggested that ETNPPL may contribute to the diagnosis, prognosis, and therapy of HCC.

## Material and Methods

### Microarray analysis

Differentially expressed mRNAs were analyzed using microarray analysis. A gene expression profile (GSE60502) was downloaded from the GEO database. The data in GSE60502 contains HCC tissues (*n* = 18) and paracancerous non-tumor tissues (*n* = 18). Differentially expressed mRNAs were acquired according to the following criterion: | log_2_ (fold change) |> 1 and *P* < 0.05.

### Functionally enrichment analysis

Gene ontology (GO) analysis and Kyoto Encyclopedia of Genes and Genomes (KEGG) pathway analysis were performed using the DAVID Bioinformatics Resources 6.8 database (https://david.ncifcrf.gov/home.jsp). *P* < 0.05 was the cutoff threshold.

### Collection of tissue specimens

A total of 30 patients diagnosed with HCC in the Affiliated Yixing Hospital of Jiangsu University were enrolled in this study. Paired HCC tissues and paracancerous tissues were collected during the surgical excision. Tissues were frozen at −80 °C for RNA isolation. The study was approved by the Ethics Committee of the Affiliated Yixing Hospital of Jiangsu University. All subjects signed the informed consents.

### qRT-PCR

Total RNA was isolated using TRIzol reagent (Invitrogen, Waltham, MA, USA). After detection of RNA concentration, 1 µg RNA was used to perform RT and qPCR using 1-step quantitative reverse transcription PCR (Bio-Rad, Hercules, CA, USA) in accordance with the manufacturer’s protocol. The expression of ETNPPL was quantified using the 2^−ΔΔCT^ approach. GAPDH was used as the normalization.

### Cell culture

HCC cell lines HuH-7, Li-7, SK-HEP-1, and normal liver cell L-02 were acquired from the Chinese Academy of Sciences (Shanghai, China). Cells were cultured in RPMI-1640 medium containing 10% FBS and 1% penicillin/streptomycin at 37 °C with 5% CO_2_. This study protocol was approved by the Ethics Committee of the Affiliated Yixing Hospital of Jiangsu University (No. 9642904843032).

### Cell transfection

Small interfering RNA-negative control (siRNA-NC) and siRNA-ETNPPL were designed and synthesized. They were transfected into HuH-7 and Li-7 cells using Lipofectamine 2000 (Invitrogen, Waltham, MA, USA) in line with the manufacturer’s protocol for 48 h.

### Western blot

After transfection, total protein was extracted using a RIPA lysis buffer. After assessing the concentration using a BCA protein assay kit (Sangon, Shanghai, China), equal protein (40 µg) was added into each lane and separated using 10% SDS-PAGE. Then the proteins were transferred onto PVDF membranes (Millipore, Burlington, MA, USA) and blocked with 5% skim milk for 1 h. The membranes were incubated with primary antibodies overnight at 4 °C, and then incubated with secondary antibody for 1 h at room temperature. Finally, the blots were visualized using ECL luminescence reagent (Sangon, Shanghai, China).

### Scratch assay

The cells in 6-well plates were incubated until 80% confluence. A scratch was made using a sterile pipette. After washing away the cell debris, the cells were incubated for 24 h. The scratch was imaged under a light microscope (Nikon, Tokyo, Japan).

### Transwell analysis

The cells were plated in the upper layer of 24-well Matrigel precoated chamber (Corning, USA). Complete medium was added to the lower layer of the chamber. The cells were cultured for 24 h and then fixed with 4% paraformaldehyde for 20 min. Lastly, the cells stained with 0.1% crystal violet were imaged and counted under a light microscope.

### Statistical analysis

The data was analyzed using GraphPad Prism 8 and displayed as mean ± SD. A student’s *t*-test and one-way ANOVA were used to evaluate the difference. The survival rate was analyzed using the Kaplan–Meier curve. The diagnostic value was analyzed by ROC curve analysis.

## Results

### Differentially expressed mRNAs in HCC

**Figure 1:**
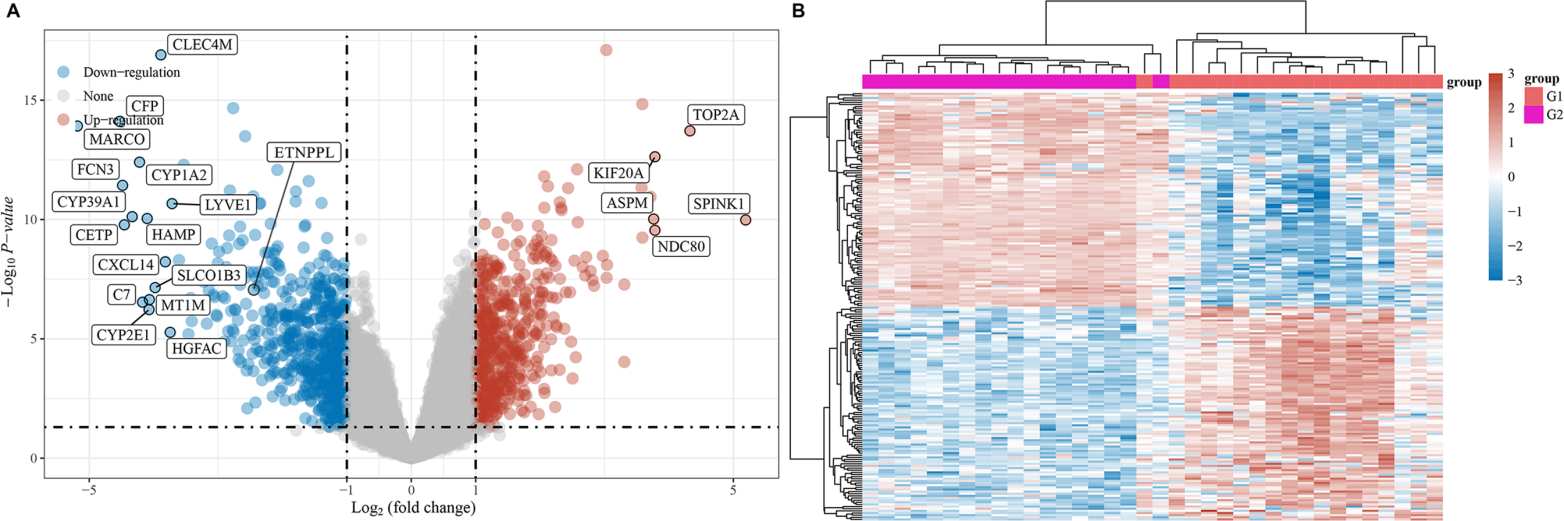
Differentially expressed mRNAs in HCC. (A) Differentially expressed mRNAs were showed using volcano plots. (B) Differentially expressed mRNAs were exhibited using heat map. Red referred to upregulated mRNAs, and blue referred to downregulated mRNAs.

A microarray assay was used to screen the differentially expressed mRNAs. The resulting volcano plots showed that numerous dysregulations of mRNAs were found between HCC tissues and paracarcinoma normal tissues (Fig. 1A). In addition, the data shown in the heat map also indicated that the expression of mRNA in HCC tissues was abnormal compared with paired adjacent tissues (Fig. 1B).

### Enrichment analysis of differentially expressed mRNAs

**Figure 2:**
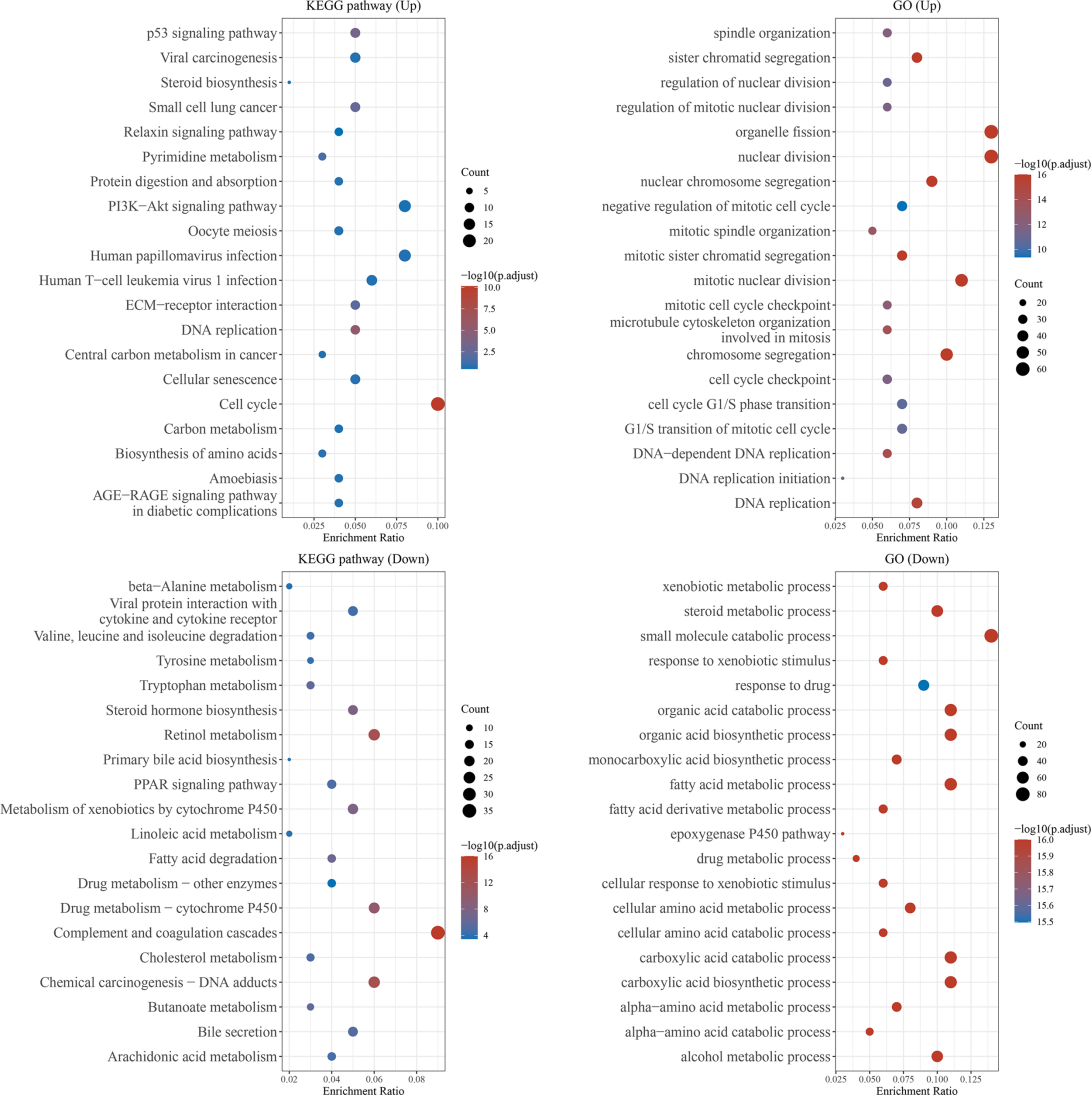
Functional enrichment analysis. The top 20 GO and KEGG pathways enriched in the upregulated or downregulated mRNAs in HCC. The *x*-axis showed the enrichment ratio, and the y-axis showed the KEGG or GO term.

We further predicted the function of upregulated or downregulated mRNAs in HCC using KEGG pathway analysis and GO analysis. The KEGG pathway analysis revealed the top 20 KEGG terms. The results indicated that both upregulated and downregulated mRNAs were enriched in several pathways, especially “metabolism”. In the top 20 GO terms, upregulated mRNAs were mainly enriched in cytomorphosis, including “organelle fission”, “nuclear division”, “chromosome segregation”, “DNA replication”, and “cytoskeleton and spindle organization”, as well as “cell cycle transition”, whereas the downregulated mRNAs were enriched in multiple metabolic and catabolic processes (Fig. 2).

### The expression of ETNPPL was decreased in HCC

**Figure 3:**
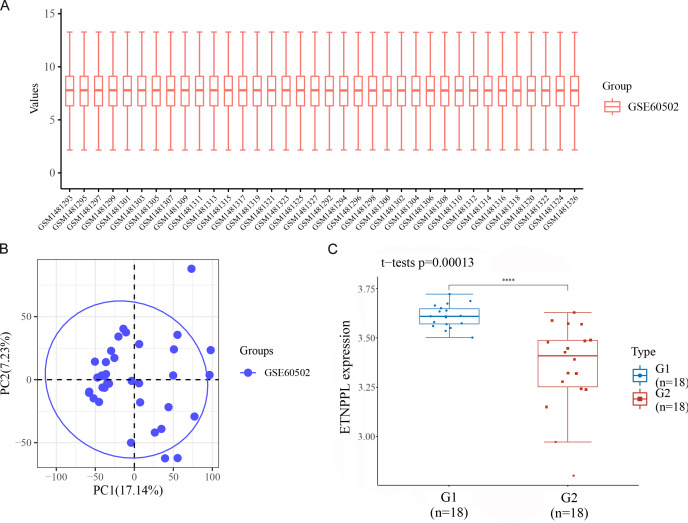
ETNPPL was decreased in HCC. The expression of ETNPPL was analyzed in tumor tissues (G1; *n* = 18) and tumor adjacent tissues (G2; *n* = 18) from the data of GSE60502 microarray. *P* = 0.00013.

Datasets were retrieved from the GEO database (https://www.ncbi.nlm.nih.gov/geo/). The keywords “hepatocellular carcinoma” and “homo sapiens” were used for the search. These datasets were then further screened according to the following criteria: (1) gene expression profiles of mRNAs; (2) samples composed of patient and normal tissues; (3) based on different data platforms; (4) the number of patient tissues and normal tissues samples was more than 10. We obtained the microarray dataset GSE60502. In all abnormally expressed mRNAs from GSE60502 microarray, we found ETNPPL was significantly downregulated in HCC tissues, compared with paracarcinoma normal tissues (Fig. 3).

### Potential diagnostic and prognostic value of ETNPPL in HCC

**Figure 4:**
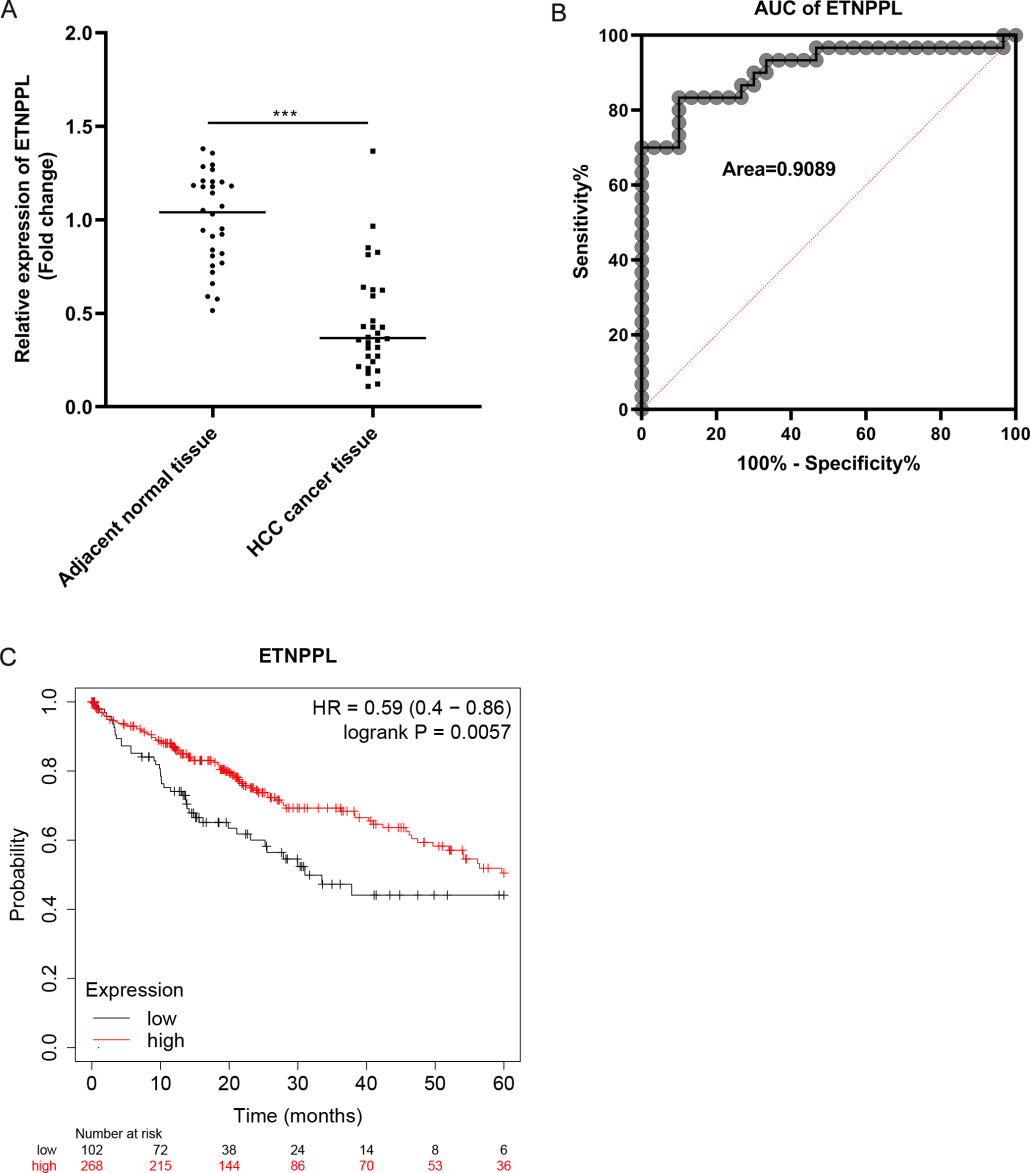
Potential diagnostic and prognostic value of ETNPPL in HCC. (A) The ETNPPL levels were tested using qRT-PCR in paired tumor tissues and paracancerous normal tissues (*n* = 30). (B) The diagnosis value of ETNPPL was assessed by ROC curve analysis. (C) Overall survival of patients with HCC was analyzed using Kaplan-Meier analysis.

As illustrated in Fig. 4A, the expression of ETNPPL was significantly downregulated in HCC tissues, compared with paracarcinoma non-tumor tissues. The potential diagnosis value of ETNPPL was assessed by ROC curve analysis. The results showed that the area under the ROC curve (AUC) was 0.9089 (Fig. 4B). The results of Kaplan–Meier analysis indicated that high expression of ETNPPL had a good prognosis (*p* = 0.0057, Fig. 4C).

### Over-expression of ETNPPL inhibits cell metastasis in HCC

**Figure 5:**
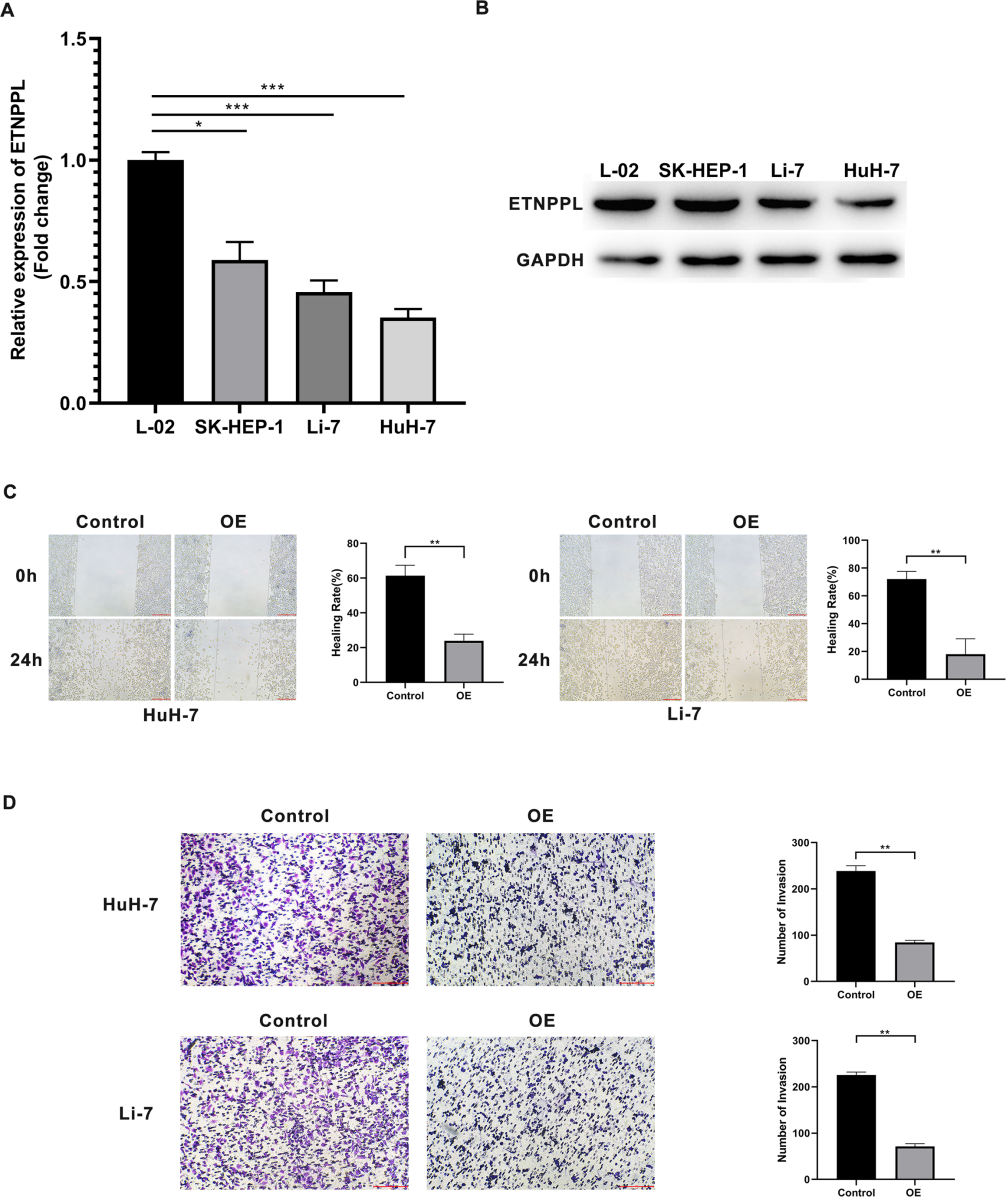
Silencing of ETNPPL facilitates cell metastasis of HCC cells. (A–B) Expression levels of ETNPPL in normal liver cells and HCC cell lines. (C) Cell migration was assessed using a scratch test. (D) Invasion transwell assay was conducted to analyze cell invasion ability.

The levels of ETNPPL were decreased in HCC cells, including HuH-7, Li-7, SK-HEP-1 cells, compared with a normal liver cell line (Fig. 5A). To explore the effect of ETNPPL on cell metastasis, ETNPPL OE-NC and ETNPPL OE were transfected into HuH-7 and Li-7 cells. The data revealed that ETNPPL was downregulated in cells transfected with si-ETNPPL (Fig. 5B). Overexpression of ETNPPL inhibited the migration and invasion of HuH-7 and Li-7 cells, compared with the ETNPPL OE-NC group (Figs. 5C and 5D).

## Discussion

HCC is a common malignant tumor of the liver, which has the biological characteristics of insidious onset, high tumor heterogeneity, difficulty in early diagnosis, and poor prognosis. The 5-year survival rate is only 12.5% (Zhou et al., 2019). Therefore, in-depth study of the molecular mechanism of HCC pathogenesis can provide ideas for finding effective potential therapeutic targets and discovering reliable diagnostic and prognostic biomarkers. In this study, we screened multiple differentially expressed mRNAs in HCC. Furthermore, we found dysregulation of ETNPPL and analyzed its diagnosis, prognosis, and treatment value in HCC.

Dysregulation of mRNAs is involved in the tumorigenesis and development of HCC. Changes in metabolism is a main feature of HCC tumors (De Matteis et al., 2018). HCC of C1 subtype showed high metabolic activity, C2 subtype showed low metabolic activity, and C3 subtype showed moderate metabolic activity  (Yang et al., 2020). Metabolic factors such as a high-fat diet, obesity, and diabetes mellitus are closely related to insulin resistance and hyperinsulinemia, which increase the expression of insulin and insulin-like growth factor-1. After binding to their receptors, they start a cascade reaction, thereby activating the downstream PI3K and MAPK signal transduction pathways. These two pathways can induce cell proliferation, inhibit apoptosis, and play an important role in the occurrence and development of HCC (De Minicis et al., 2014). At present, many clinical parameters of liver function reflect metabolic alteration. Similarly, in this study, we acquired multiple abnormally expressed mRNAs in HCC using microarray analysis. Furthermore, we predicted that the upregulated and downregulated mRNAs are closely related to metabolism processes. The data suggested that these abnormally expressed mRNAs are related to the abnormal metabolism in the liver.

Previous studies have reported that several mRNAs serve as diagnostic biomarkers. For example, serum HITIH4 has the value of early diagnosis of HCC (Li et al., 2018). Lipocalin-2 is highly expressed in the serum of patients with HCC, and is a biomarker for diagnosis of HCC (Barsoum, Elgohary & Bassiony, 2020). Arginase-1 is also a biomarker of HCC that helps distinguish primary and metastatic tumors (Sang et al., 2015). Additionally, prognosis is associated with diagnosis. Improving our diagnostic toolset can enable earlier identification of the disease and improve patient prognosis. In recent years, a variety of prognosis biomarkers of HCC have been discovered. For instance, low levels of CCL14 are related to a worse prognosis (Gu et al., 2020). Zhao et al. (2021) found that CDC20 is highly expressed in liver cancer tissues, which may regulate the proliferation and radiosensitivity of liver cancer cells with P53 mutation through the Bcl-2/Bax pathway and is a marker of poor prognosis in HCC patients. YTHDF1 is a possible prognostic marker of HCC, which when highly expressed indicates poor prognosis (Zhao et al., 2018). However, early screening for HCC remains difficult so far. Identifying more novel markers is required.

Phosphoethanolamine is an important raw material for the synthesis of phosphatidylcholine and phosphatidylethanolamine. As the two types of phospholipids that occur in the highest concentration in the cell membrane, abnormalities in phosphatidylcholine and phosphatidylethanolamine can lead to abnormal conduction of cell signaling pathways (Eyster, 2007). The possible mechanism is that they directly affect the activity of membrane receptors, leading to abnormal production of second messengers. The expression product of ETNPPL has recently been found to possess the phosphatase activity of phosphoethanolamine (PEA), capable of irreversibly degrading PEA to yield phosphate, acetaldehyde, and ammonia. In recent years, a number of studies have found that synthetic PEA has inhibitory effects on a variety of tumor cells. For example, in renal cell carcinomas, PEA can inhibit lung metastasis of tumor cells, and down-regulate a series of tumor-related proteins such as cyclin D1, vascular endothelial growth factor receptor-1 (VEGFR1), *etc*. (Luna et al., 2018). PEA induced G2/M cell cycle arrest, abnormal mitochondrial membrane potential, and caspase-3-dependent apoptosis in melanoma cells (Ferreira et al., 2012). Therefore, we hypothesized that ETNPPL might be an important gene in the regulation of cellular phospholipid synthesis, and ETNPPL knockdown in tumor cells would lead to the accumulation of endogenous PEA, thereby acting as a tumor suppressor. At present, there are few studies on ETNPPL, mainly focusing on cancer and multiple types of psychiatric disorders  (Shao & Vawter, 2008; Deng et al., 2020; Stankiewicz et al., 2014). Studies have shown that ETNPPL is downregulated in malignancies, including colorectal cancer, gastric cancer and pancreatic cancer, and low expression of ETNPPL indicates poorer prognosis (Ding et al., 2016; Lucas et al., 2005). In HCC, Ding et al. (2016) reported that ETNPPL expression is also decreased. Downregulation of ETNPPL is related to unfavorable prognosis and facilitates lipogenesis of HCC cells. However, whether ETNPPL has diagnostic value is still unclear. In this study, we also found ETNPPL levels were reduced in HCC. The AUC was 0.9089, and low ETNPPL referred to poor prognosis. Moreover, low levels of ETNPPL were associated with advanced tumor node metastasis classification (TNM) stage, poor grade, and tumor metastasis. The findings suggest that ETNPPL has potential diagnostic and prognostic value.

Finally, we investigated the biological behaviors of ETNPPL in HCC cells *in vitro*. Over-expression of ETNPPL inhibited the migration and invasion of tumor cells, suggesting that ETNPPL plays a tumor suppressive role. The results showed that ETNPPL may be a novel target for HCC therapy.

In summary, ETNPPL was downregulated in HCC. Downregulation of ETNPPL was related to poor prognosis and promotion of cell metastasis. These findings suggest that ETNPPL is a promising biomarker of diagnosis and prognosis and can be used as a potential therapeutic target of HCC.

## Ethics Approval

This study protocol was approved by the Ethics Committee of Yixing Fourth People’s Hospital (No. 9642904843032). Written informed consent was provided prior to the study.

##  Supplemental Information

10.7717/peerj.15834/supp-1 Supplemental Information 1 Original bands

10.7717/peerj.15834/supp-2 Supplemental Information 2 Figure 4 A original data

10.7717/peerj.15834/supp-3 Supplemental Information 3 Figure 5A PCR data

10.7717/peerj.15834/supp-4 Supplemental Information 4 Figure 5B original bands

10.7717/peerj.15834/supp-5 Supplemental Information 5 Figure 5c original data

10.7717/peerj.15834/supp-6 Supplemental Information 6 Figure 5D original data

## Additional Information and Declarations

### Data Availability

The following information was supplied regarding data availability: The raw data are available in the Supplemental Files.
